# Ethical issues concerning UK veterinary surgeons practicing in equine sports medicine

**DOI:** 10.1111/evj.14204

**Published:** 2024-07-20

**Authors:** Kate Allen, Mike King, Lynley Anderson, Siobhan Mullan

**Affiliations:** ^1^ University of Bristol Langford UK; ^2^ University of Otago Dunedin New Zealand; ^3^ University College Dublin Dublin Ireland

**Keywords:** ethics, horse, sports medicine, veterinary

## Abstract

**Background:**

The ethics of equine sports medicine is a complex subject that is currently understudied. It combines veterinary ethics, sports ethics and associated regulation. Equine sports medicine may raise unique ethical issues and combines common ethical issues in ways distinct from other forms of veterinary medicine.

**Objectives:**

The purpose of this research was to identify and describe ethical issues concerning United Kingdom (UK) veterinary surgeons arising within the practice of equine sports medicine.

**Study design:**

Survey.

**Methods:**

An online questionnaire was distributed to UK veterinary surgeons via veterinary organisations and veterinary social media. Responses to questions were collated and descriptive analysis performed. Open ended responses were analysed thematically.

**Results:**

Ninety‐seven respondents completed the questionnaire. The most commonly identified ethical challenges were the conflicts of interest and the pressures faced by the veterinary surgeon. The primary competing interest was balancing the horse's health and welfare with client wishes for the horse to continue in training and competition. Specific ethical challenges were identified; these related to competition integrity, medication control and prohibited substances, treatment evidence and acceptability, among others.

**Main limitations:**

As anticipated with the use of a questionnaire, the responses did not provide in‐depth information about an individual veterinary surgeon's experiences, however, it did provide evidence of the extensive range of issues and concerns facing this group. There is also potential for response bias, whereby respondents may have provided answers they perceived were ethically desirable.

**Conclusions:**

This is the first empirical study that explores the ethical issues faced by equine sports medicine veterinary surgeons and has identified wide ranging concerns that demand further study. Areas which may pose reputational risk to equestrian sport, or the veterinary profession were identified. Governing bodies should consider how to improve support for veterinary surgeons facing ethical challenges, as for some, these cause moral distress and may impact retention within the profession.

## 1 INTRODUCTION

The practice of equine sports medicine is complex. It combines the ethical complexities of commercial veterinary practice and human sports medicine, superimposed on the ethically contested background of the use of horses for sport. The presence of a human rider is a further complication. The ethics of equine sports medicine is, therefore, an area which deserves distinct consideration. A recent scoping review of equine sports medicine ethics identified, explored and reported some of these ethical challenges.^1^ Along with a need for more ethical analysis of these challenges, a key finding was the lack of empirical research identifying ethical concerns faced by equine sports medicine veterinary surgeons (ESMVS).^1^ There is an absence of objective information on how conflicting health, welfare and performance issues are managed in equine veterinary practice and regulated in equine sport. Equine sports medicine has developed as a specialism over recent decades, and this should be matched with research‐driven policy to understand and adapt to ethical challenges as they arise. There is a need for further empirical research, ethical reasoning, and ethically informed evidence‐based policy development in this area.

Although we may hypothesise about the concerns of veterinary surgeons in the United Kingdom (UK), a questionnaire for veterinary surgeons in clinical veterinary practice is required to better identify these. Empirical research can reveal barriers to effective care, their harms and benefits, how veterinary practitioners manage these, and the effectiveness of these strategies. This provides data to inform ethical analysis which aims to provide a well‐reasoned account of ethical and professional practice. This will enable governing bodies to ensure processes are in place to support individual veterinary surgeons who are striving for the highest levels of ethical and professional conduct in equine sports medicine. It would also raise awareness of specific veterinary practices which are considered ethically challenging by some and enable governing bodies to provide further guidance or regulations around these. Policy can be developed from this to coordinate and ensure consistency in practice. When well‐designed and operationalised, this can protect the welfare of horses, riders, and veterinary surgeons, as well as the integrity and social licence of the sport and the profession.

The intention of this study was to identify and map these ethical issues by canvassing equine veterinary surgeons working within the area of equine sports medicine in the UK.

## 2 MATERIALS AND METHODS

An anonymous online questionnaire was designed to explore and identify ethical issues facing veterinary surgeons who deliver clinical practice in the field of equine sports medicine in the UK. For the purposes of this study ‘equine sports medicine’ referred to a veterinary surgeon advising on ‘*the structural, physiological, medical, surgical and welfare needs of racehorses and sport horses and the restoration of normal form and function after injury or illness*’ (adapted from American College of Veterinary Sports Medicine and Rehabilitation^2^). The questionnaire was based on a similar survey of sports medicine doctors,^3^ adapted to include areas already raised as potential ethical concerns.^4^ The questionnaire was reviewed by five council members from the British Equine Veterinary Association (BEVA), and the Research and Education Officer from a Welfare Charity, and further revised. The complete set of questions featured in the questionnaire, along with their respective answer options, including both structured choices and opportunities for open text responses, is provided as a supplementary item (Questionnaire S1). The questions encompass various aspects of the respondent's clinical veterinary work, ethical challenges, conflicts of interest, opinions on medications and treatments, clarity of obligations set by governing bodies, priority areas and considerations regarding reputational risk. The questionnaire entitled ‘Equine Sports Medicine Ethics’ was open for completion for a 10‐week period between September and November 2021. Equine veterinary surgeons practicing in the UK were eligible to respond and the questionnaire was promoted by BEVA to its members, via veterinary social media posts, and to the membership of an online continuing professional development provider.

Answers to open‐ended questions were analysed thematically. Quotes are used for two purposes throughout this paper; first, to capture the particular theme expressed by a number of respondents, and second, to ensure that the voices of the respondents are maintained and expressed.

Data are presented as counts and percentages. A Wilcoxon signed‐rank test was used to assess if there were differences in whether respondents perceived the duties/obligations of veterinary surgeons to be well defined by the governing body during the competition period compared with the between competition periods. A *p* value of <0.05 denoted statistical significance.

## 3 RESULTS

### 3.1 Respondents

Ninety‐seven respondents completed the questionnaire, however, not all respondents answered all questions. Respondents (*n* = 93, 96%) varied as to the proportion of equine sports medicine that comprised their veterinary work: for 21.5% of respondents 25% of their work was dedicated to clinical equine sports medicine, 28% were in the 25%–49% category, 28% in the 50%–74% category and 22.6% did more than 75% of their work within sports medicine. Fifty‐seven respondents (59%) had post‐graduate veterinary qualifications.

A large proportion (*n* = 62, 64%) of respondents also provided veterinary services at competitions/races. Respondents provided veterinary services to horses in a variety of equestrian disciplines with considerable variation as to the proportion of time working in each discipline. The mean proportion of time spent in each discipline was: showjumping 28%, eventing 25%, dressage 24%, racing 22%, polo under 2%, endurance and driving under 1%.

### 3.2 Stakeholder responsibility

**FIGURE 1:**
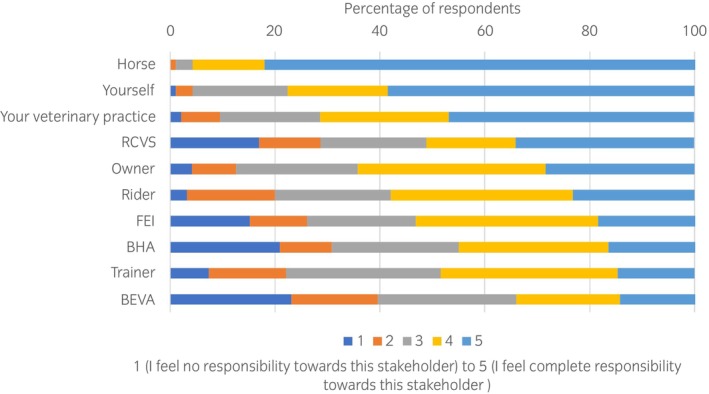
Equine veterinary surgeons' (*n* = 95) self‐reported responsibility to various stakeholders when practising in equine sports medicine.

When respondents (*n* = 95, 98%) were asked the degree of responsibility they felt towards varying stakeholders, the highest was to the horse, with 82.1% respondents selecting ‘I feel complete responsibility towards this stakeholder’ (Figure 1). This was followed by 58.5% selecting complete responsibility to themselves and 46.8% selecting complete responsibility to their veterinary practice, followed by the Royal College of Veterinary Surgeons (RCVS) at 34.0%, owner, rider and trainer at 28.4%, 23.2%, 14.7% respectively, and other governing bodies and organisations (Federation Equestre Internationale [FEI] [18.5%], British Horseracing Authority [BHA] [16.5%], BEVA [14%]).

### 3.3 Ethical challenges

There were 86 responses (89%) to the question ‘What (if any) do you think are the ethical challenges facing veterinary surgeons who provide equine sports medicine services?’, with many respondents listing several ethical challenges. Three responded that there were no ethical challenges. Responses were categorised into general ethical themes, leaving a small remainder of specific issues.

The most common general ethical challenge covered conflicts of interest, pressures on the veterinary surgeon and competing interests, and was stated or alluded to by 50 (52%) respondents. The primary area was balancing the horse's health and welfare with client wishes, in particular for the horse to compete. Responses included: ‘*Finding the correct balance between the horse's welfare and it continuing to compete at the highest level*’, ‘*managing conflicts of interest between stakeholders' aspirations and the welfare of the horses*’, ‘*placing horse welfare second to performance*’. A common example of this was conflicts between ongoing competition and the need for rest or retirement: ‘*Owners requesting ongoing treatment and management of injuries in order to allow the horse to continue competing at a high level, where it might be more appropriate to drop to a lower level/retire the horse*’.

Some respondents noted client demands around treatment: ‘*meeting the needs of the client without allowing them to dictate treatment*’ with others noting there was pressure to provide a ‘*quick fix*’ and to ‘*patch up*’ a horse for it to compete or be sold.

Some respondents identified conflicts with competition integrity: ‘*pressure to accept horses* [in competitions] *that are not fit to compete*’, ‘*treating horses to improve performance but adhering to the ethos of clean sport*’, ‘*ensuring that sports are conducted transparently with the horse's welfare paramount*’.

Intra‐articular medications (excessive use, inappropriate use, pressure to use) were specifically mentioned by 19 respondents. A high prevalence of lameness, or chronic lameness was mentioned by 10 respondents. Furthermore, a lack of owner/trainer recognition of lameness, and disregard of lameness was mentioned: ‘*chronic lameness and owners expecting to continue competing regardless*’. The possibility of veterinary surgeons losing objectivity around normal function, health and welfare was also mentioned in the context of lameness assessment: ‘*I often wonder whether our eye is so skewed by looking at chronically lame racehorses all the time that the balance of judgement on what is acceptable has lost its way somewhat, probably in the most part due to pressure from trainers*’.

Upper airway surgeries or ‘*wind ops*’ were mentioned by 11 respondents.

Lack of evidence or scientific basis for treatments was mentioned by nine respondents: ‘*treatments done not always with clinical indication and no real scientific basis*’.

Administering treatments without appropriate prior diagnostic investigations was mentioned by seven respondents. Often this was clarified as a lack of diagnostic investigation of lame horses prior to treatment.

### 3.4 Most challenging situation

When asked to describe the circumstances of the most challenging type of situation, more than half of the 81 (84%) respondents identified situations similar to their answer to the preceding question (which was What [if any] are the ethical challenges facing veterinary surgeons who provide equine sports medicine services?). These included pressure for the horse to compete, pressure from the client, health conditions affecting horse welfare, provision of medications too close to competition and excessive or inappropriate intraarticular medications.

Responses included:

being asked to medicate/administer a drug and not put it on the clinical record as the rider/owner knows it isn't allowed that close to competition,

dealing with lame horses that are required by the owner/rider/trainer to compete come what may.

Other themes mentioned were euthanasia and the challenges of veterinary provision at the racetrack:

Conflict management on race day when a horse is considered unsuitable to race, contrary to the view of the trainer. Pressure to allow horses to race that are ‘poor movers’, which in my opinion is a euphemism for a lame horse that we allow to cloud our judgement. Treading the line of acceptability of discomfort for the horse is hard. I sometimes wrestle with communicating clearly and effectively to trainers that just because an animal is performing well doesn't mean it isn't in pain sufficient to compromise its welfare over a period of time,

rapid decision making about the withdrawal of horses in pressurised situations such as the starting stalls can also be a challenge,

attending racecourse casualties under the full view of onlooking public and television/press.

When asked how commonly their most challenging situation arose, the most frequently chosen response (53.5%) was a few times a year, with a further 29.1% of respondents choosing ‘monthly’ or ‘weekly or more’.

### 3.5 Pressures and conflicts of interest

**FIGURE 2:**
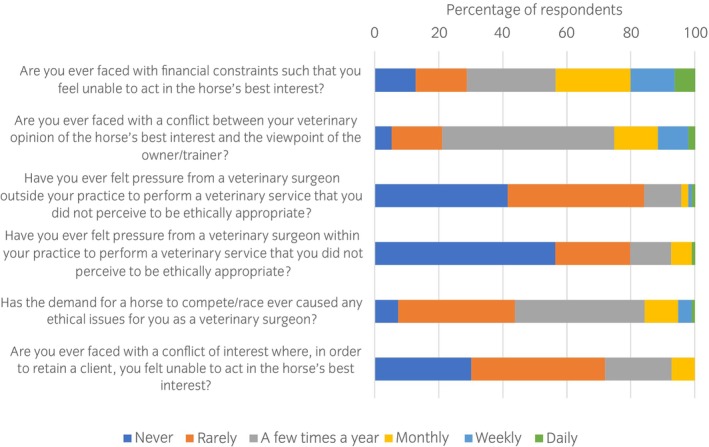
Equine veterinary surgeons' (*n* = 94–96) responses to a series of questions designed to identify the frequency with which various factors lead to pressures or conflicts of interest when providing equine sports medicine veterinary services.

The questions that were designed to identify factors which may lead to pressures or conflicts of interest when providing equine sports medicine veterinary services were answered by 94–96 (97%–99%) respondents (Figure 2). Financial constraints and differences of opinion with the owner/trainer were most commonly encountered.

Respondents had the opportunity to identify other areas of conflict of interest/pressure. There were 44 responses. Many provided examples of a similar conflict to those above, that is, pressure from an owner to perform a treatment or sign a declaration, pressure from a senior veterinary surgeon to perform a treatment, lack of finance for appropriate diagnostics etc. Other conflicts identified included competition between veterinary practices and lack of time. Interestingly, there were several responses in the theme of ‘*pressure for good outcome*’, ‘*reputational pressure*’, ‘*fear of failing to get correct diagnosis*’. Other areas identified were pressure around the trot‐up [veterinary inspection for lameness], and pressures/conflicts around pre‐purchase. Other conflicts were performing procedures on horses that were then used for breeding, conflicts around rehabilitation such as box‐rest, and that choice of medication may be based on withdrawal times rather than choosing the most appropriate drug. Other concerns included ‘*offering services for commercial reasons in the absence of scientific evidence*’.

### 3.6 Medications and treatments

Specific questions were asked around medication control and prohibited substances. When asked ‘Are there any medications or other substances that are not prohibited which you think should be?’ the majority (*n* = 32) of the 43 respondents chose ‘no’. Specific responses included: sarapin (*n* = 2), tiludronic acid (*n* = 1), pentosan polysulfate sodium (*n* = 1), bisphosphonates (*n* = 1), blisters (*n* = 1), omeprazole (*n* = 1), over the counter calmers (*n* = 1) and any prescription medicine apart from antimicrobials (*n* = 1). There were two more general comments which discussed any substance being used for competitive advantage:

Anything administered to a horse that is given to improve performance should be prohibited, why are there ‘therapies’ allowed to be administered to horses at competitions, if they are being done it's to improve performance so they should all be banned, to not do so is not in keeping with promoting horse welfare.

When asked ‘Are there any medications or other substances that are currently prohibited which you think should not be?’ there were 50 respondents. ‘No’ was selected by 17, and varying medications were listed by others: omeprazole *n* = 10, pergolide *n* = 11, altrenogest *n* = 2, frusemide *n* = 2, methotrexate *n* = 1, procaine penicillin *n* = 1, non‐steroidal anti‐inflammatory drugs *n* = 1, multivitamins *n* = 1, canagliflozin *n* = 1, intra‐articular anabolic steroids *n* = 1 and naturally occurring nutraceuticals (*n* = 1).

When asked whether there were any permitted veterinary treatments or procedures that respondents consider ethically unacceptable, there were 41 (42%) responses. ‘Wind ops’ was mentioned 14 times, with soft palate cautery in particular mentioned 9 times. Tendon firing was mentioned 13 times. ‘*Firing*’ without clarification of the structure involved was mentioned a further six times, blistering six times and neurectomy or neurectomy/fasciotomy seven times. Shockwave, joint injections, kissing spine surgery, treatments with lack of evidence of efficacy, acupuncture, laser and chiropractic were also mentioned at least once.

When asked whether there were permitted veterinary treatments or procedures that respondents thought were used excessively within equine sports medicine, such that they may not be in the horse's best interest, the two clinical areas with the most answers were joint injections (*n* = 27), and upper airway surgery (*n* = 18). Other responses were shockwave therapy (*n* = 5), denervation (*n* = 4), IV fluids (*n* = 2), kissing spine (*n* = 4), stem cells (*n* = 1) and ‘*loads*’ (*n* = 1).

### 3.7 Sensitive information

**FIGURE 3:**
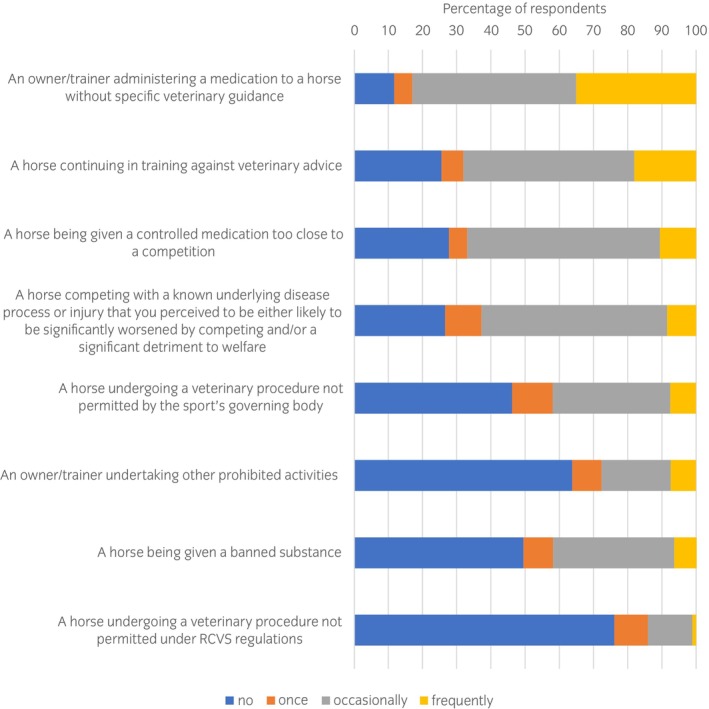
Equine veterinary surgeons' (*n* = 94) self‐reported frequency of exposure to different types of sensitive information in the equine sports medicine context.

It was anticipated that veterinary surgeons may at times be aware of highly sensitive information and a series of questions was asked to explore this further (Figure 3). Of the *n* = 94 (97%) respondents, 35% reported frequently encountering ‘*owners/trainers administering a medication without veterinary advice*’ and a further 48% encountered this occasionally. At least 50% of respondents also reported encountering ‘*a horse continuing in training against veterinary advice*’, ‘*a horse being given a controlled medication too close to a competition*’ or ‘*a horse competing with a known underlying disease process or injury that you perceived to be either likely to be significantly worsened by competing and/or a significant detriment to welfare*’ either frequently or occasionally. Respondents were aware of one or more occasions where a horse underwent a procedure not permitted by a sporting governing body (*n* = 54%), and a horse being given a banned substance (*n* = 51%). Twenty four percent of respondents were aware of one or more occasions where a horse underwent a veterinary procedure not permitted by the RCVS, and 36% were aware of prohibited activities performed by an owner/trainer. The majority of respondents had not reported these situations to any governing body, reasons included: ‘*insufficient evidence*’, ‘*risk of losing employment*’, ‘*risk of losing client*’, ‘*it's too common to attempt to deal with single handedly*’, ‘*not permitted under client confidentiality*’. Six respondents said that they had reported the situation to a governing body.

### 3.8 Regulatory organisations

**FIGURE 4:**
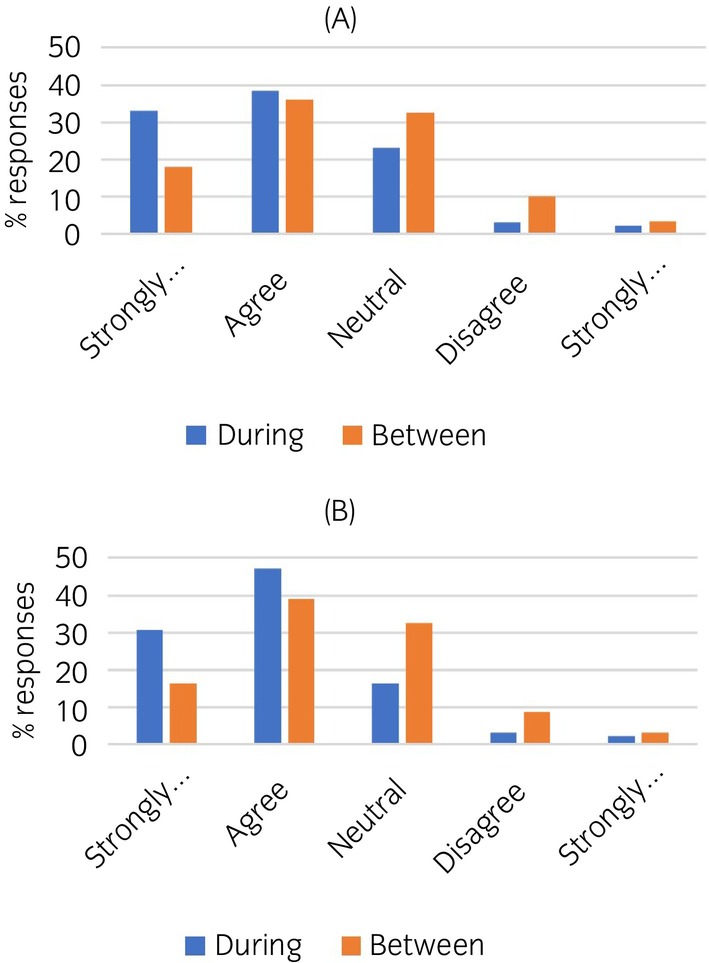
Graphs showing the significant difference in perceived clarity of regulation during the competition period compared with the between competition period for (A) British Horseracing Authority (*p* < 0.001) and (B) Federation Equestre Internationale (*p* < 0.001).

A series of questions was asked that aimed to identify whether respondents perceived any differences in whether the duties and obligations of veterinary surgeons were well defined by the regulatory body, comparing the during competition/race period with the between competition/race period (Table 1). There was no significant difference in opinions when considering the RCVS, but for the sporting governing bodies (BHA and FEI) there were significant differences in opinions with a shift towards less agreement that duties/obligations are well defined in the between competition period (Figure 4).

**TABLE 1:** Equine veterinary surgeons' perceived clarity of regulation during the competition period compared with the between‐competition period.

	During competitions/races, the duties and obligations of veterinary surgeons are well defined by the regulatory body					Between competitions/races, the duties and obligations of veterinary surgeons are well defined by the regulatory body					*p*‐Value
	Strongly agree	Agree	Neutral	Disagree	Strongly disagree	Strongly agree	Agree	Neutral	Disagree	Strongly disagree	
RCVS	15.2	33.7	32.6	14.1	4.3	16.7	36.7	32.2	11.1	3.3	0.14
BHA	33.0	38.5	23.1	3.3	2.2	18.0	36.0	32.6	10.1	3.4	<0.001
FEI	30.8	47.3	16.5	3.3	2.2	16.3	39.1	32.6	8.7	3.3	<0.001
HPA	12.5	20.0	58.8	5.0	3.8	7.4	14.8	63.0	11.1	3.7	0.05

*Note*: Table shows percentage responses. Signed‐rank test compared the two time periods. (Number of respondents *n* = 93 RCVS, *n* = 91 BHA/FEI, *n* = 80 HPA.)

### 3.9 Reputational risk and priority areas

Many of the responses and themes in the final set of open‐ended questions were similar. ‘Which equine sports medicine ethical challenge (if any) do you think is the highest priority for the industry to address?’, received 70 (72%) responses. There was a strong focus on the high prevalence of lameness, and of other diseases (e.g., gastric ulceration), and how to minimise injuries and fatalities. Training methods and the high training and competition loads were also discussed:

Balancing the stresses and strains of training placed on the equine athlete with their welfare ‐ what is acceptable for us to ask of them? Human athletes report being in almost inevitable discomfort during training and competition at the highest levels and require significantly more medical intervention (particularly from an orthopaedic perspective) than lay members of the public. Is it acceptable that we ask the same of a horse? How do we balance that with the general public's perception of the use of horses in sport and the maintenance of our social licence?

Other themes focused on a lack of pain/lameness recognition and provision of treatments without clinical indication or appropriate diagnostics or which lack evidence of efficacy. Other responses mentioned treatments being administered that were not under veterinary guidance and client administration of medications. Excessive or inappropriate use of ‘wind ops’, joint injections, medications, neurectomy, obesity, and hypersensitisation were all mentioned. The importance of prioritising welfare was discussed, along with high wastage [horses retired from racing/competition] and quality of life in retirement.

Two questions focused on the reputational risk of certain practices. First, respondents were asked to list any elements of equine sports medicine that they believed posed a reputational risk to equestrian sport (i.e., elements that may cause significant public concern). In total there were 63 (65%) responses, some of which were not veterinary specific (e.g., racing, polo, endurance, whip use). The responses relevant to sports medicine, again focused on treatments without clear indication, treatments without prior diagnostics, intra‐articular medications, wind ops, firing, neurectomies, IV fluids, injury rates, wastage, disease rates (e.g., gastric ulceration/lameness), fatalities at the racetrack, euthanasia, medication control (lack of) and doping. The only comment that had not previously been raised elsewhere in the responses was ‘the lack of veterinary supervision at some sports, e.g., polo’.

Second, respondents were asked to list any elements of equine sports medicine that they believed posed a reputational risk to the veterinary profession. There were 49 (51%) responses, with many similarities to the previous question. The responses included intraarticular medications, firing, ‘wind ops’, neurectomies, kissing spine surgery, treatments with little evidence of efficacy, medication usage including banned substances, antibiotic usage, excessive treatments and ‘*maintenance*’ therapies.

There were 59 (61%) responses listing areas respondents felt would be highest priority for further research aimed at improving the welfare of competition horses/racehorses. There was a focus on particular diseases, procedures and treatment efficacy which included airway surgeries, intra‐articular disease, tendon injuries, catastrophic injuries, exercise induced pulmonary haemorrhage, suspensory ligament desmitis and overriding dorsal spinous processes. There was also a strong focus on improvement of injury reporting during training, risk modelling for injury and pre‐race screening. Husbandry practices, welfare assessment, assessment of equine emotional distress, training and track surfaces research and the reporting and tracing of medications and procedures were all mentioned. Finally, how to improve veterinary ethics in the UK was raised.

The final question enabled respondents to raise any other issues of concern to themselves as an equine veterinary surgeon in this area. Some of the topics included among the 36 (37%) responses, already feature elsewhere in the results such as specific procedures and conflicts of interest, ‘*Managing the welfare of the horse in competition animals is challenging as multiple interested parties are present and the horse's voice is the vet and the pressure on them is immense*’. Comments that may not be well represented elsewhere were grouped into those relating to the veterinary profession and those relating to competition. Comments relating to the veterinary profession included: ‘*Essential to have/continue to have a very strong and effective regulatory authority to prevent vets acting in an unethical manner*’, ‘*dishonesty of vets in carrying out treatment that may be prohibited/unethical because they don't want to lose a client. We need to have a backbone, stick together and work for the horse's best interests*’, ‘*a lack of accountability by the veterinary profession for the impact on the legacy of the sport, i.e., their behaviour is too short‐termist*’, ‘*The equine veterinary profession is experiencing difficulty attracting and retaining young veterinarians due to the ethical issues stated above and the dysfunctional business model in which the trainer directs the veterinarian and has the power to fire the vet if he or she does not acquiesce*’ and finally ‘*being grossly/obscenely underpaid*’. A few respondents (*n* = 5) commented that when multiple veterinary surgeons were involved in the care of a horse issues can arise; that international ESMVS were entering and working in the UK despite not having RCVS registration, and that sports medicine veterinary surgeons might be a limited‐service provider and might not contribute to out‐of‐hours/emergency cover or communicate well with the primary veterinary practice, leading to ‘*lack of transparency, traceability of records and lack of responsibility*’ by some sports medicine veterinary surgeons.

Further comments, not mentioned elsewhere, relating to competition/sport include: ‘*there needs to be a way for judges to disqualify overtly lame horses from competition and there needs to be precedent set to make this ok*’; ‘*there needs to be improved drugs testing outside of the racing industry*’; ‘*the failure of the* [named governing body] *to act sensibly in identifying real horse welfare issues rather than perceived*’; ‘*in need of more official and veterinary personnel during competitions*’, and ‘*the idea of* “*clean sport*” *is an ideal but a million miles away from human athletes who can be dope tested at any point in training or competition*’.

## 4 DISCUSSION

Among a wide range of ethical concerns identified, these results confirm that veterinary surgeons have a strong sense of responsibility to the horse, which aligns to the RCVS Code of Professional Conduct that ‘*Veterinary surgeons must make animal health and welfare their first consideration*’. However, it is clear that ESMVS regularly face conflicts in their role that make it difficult to meet the goal of putting equine health and welfare first, revealing a complex environment with multiple loyalties, competing demands and subsequent obligations. We define competing obligations as occurring when the satisfaction of one obligation entails the sacrifice of another. These competing demands or obligations include meeting the welfare needs of the horse, the competition demands of the owner and/or trainer and the ESMVS's own personal business interests, as well as the professional expectations set out by the governing bodies.

Competing obligations also arise as a key issue for sports medicine doctors who face similar pressures to return an athlete to sport more quickly than is medically indicated, creating a tension between the long‐term welfare of the player/patient and the demands of the coach, the player or fans.^3^ In human sports medicine, the athlete may also be vulnerable because of their age, their sense of obligation to their team or coach, and/or their desire to retain a contract and this may render them compliant with training regimes and treatments that may not be in their best interest. Some similarities may be drawn between equine clinical decision‐making and clinical decision‐making for child athletes,^5^ where decisions and consent are provided by a parent. However, children generally gain competence over the course of their athletic careers. A key difference for ESMVS is the extreme vulnerability of the patient who lacks agency and, in extreme cases, is expendable.

Having to meet the interests of others (owners, riders, trainers) when providing care is common in many aspects of veterinary medicine and is a component of shared decision‐making. However, the pressure for a horse to compete and the conflicts of interest that may arise in this situation were the primary challenges identified by respondents (and previously identified in an equine sports medicine scoping review^1^). This can occur when the owner's interest conflicts with the best interests of the horse. Such conflicts are common in veterinary practice, however the nature and degree of the conflict in equine sports medicine makes this distinct in its prevalence and severity. At stake for the veterinary surgeon, trainer, owner, and rider will routinely be professional success, both reputational and financial. Furthermore, the context of equine sports is competitive, introducing a potentially overriding reason to seek positional advantage over competitor equine athletes. This is a situation of competitive escalation, which marks it as distinct from the routine stakeholder conflicts in veterinary care. Challenges for veterinary surgeons in clinical decision‐making should be supported by the development or use of ethical codes,^6^, ^7^ ethical standards in decision‐making^8^ and ethical frameworks.^9^, ^10^ These will also require ethical reflection that considers the particular features of clinical situations, allowing nuanced case‐by‐case decision‐making.

Trying to balance competing obligations can lead to moral distress for ESMVS. We define moral distress as when the ESMVS knows the appropriate action to take but is unable to do so due to some constraint. These constraints can include competing obligations, professional or personal values. The questions were not set up to fully explore the moral distress experienced by the respondents, but words such as ‘*hard*’, ‘*wrestle with*’, ‘*difficult*’, ‘*challenging*’ and ‘*horrified*’ were used by respondents along with the repeated references to feeling pressured, for example, ‘*the pressure on them* [veterinary surgeons] *is immense*’. These ethical issues and the resulting moral distress may influence veterinary students considering a career in equine sports medicine, as well as negatively affecting retention within the profession.

Our results also revealed pressures upon veterinary surgeons not previously identified in the scoping review.^1^ In particular, this includes providing veterinary services at competitions/races, which is a pressured environment requiring rapid decision‐making in view of public and spectators. Second, the responsibility of providing a good outcome for the horse was a pressure not previously identified. This can come from having to meet expectations of owners and trainers while also meeting an ESMVS's personal values.

When rating the degree of responsibility veterinary surgeons feel towards various stakeholders, the responsibility towards the RCVS was lower than may be expected, considering this is the regulatory authority in the UK. This may indicate that the weight of competing obligations are such that professional obligations are diminished. Regulations that can be used to support and protect an ESMVS from undue influence should be considered.

Some of these findings will be concerning for governing bodies. Of particular concern is that half of all veterinary surgeons were aware of a horse being given a banned substance. Although the questionnaire did not explore whether veterinary surgeons had any involvement in this practice, the finding does indicate the presence of doping in equestrian sport. A quarter of veterinary surgeons were aware of horses being subjected to procedures banned by the RCVS and over half were aware of a horse undergoing a veterinary procedure that is not permitted by a sporting governing body thus suggesting that the governing bodies and ESMVS at the coalface are not well aligned. A high proportion of participants knew that a horse had received a controlled medication too close to a competition, that a horse continued in training against veterinary advice and that a horse had competed with an underlying disease process that was likely to be worsened by competition. Although this survey offered anonymity, underreporting of sensitive information may still occur.^11^ It appeared that few veterinary surgeons reported illicit practice when they were aware of it. The reasons were multifactorial and include conflict with client confidentiality. Similarly, sports doctors will also often choose to keep sensitive information confidential, likely from a perceived overriding responsibility to the athlete.^12^ The challenges of confidentiality could be eased by clearer guidance on the professional expectations when faced with these situations.^12^ There appears to be a lack of clarity on the process of reporting and where the veterinary surgeon's professional obligations lie. Some respondents were concerned that there was a lack of penalty for performing a banned surgical procedure. Collectively, these concerns appear to indicate that the professional bodies may lack safe reporting mechanisms and deterrence.

The survey confirms that at least some UK veterinary surgeons have ethical concerns about the use of particular treatments. Those that were most commonly identified as being ethically unacceptable were: intraarticular medications, upper airway surgeries and firing. The high prevalence of lameness in the performance horse during training and competition, is clearly a concern for many. Thus far, there has not been a format for these concerns to be raised, discussed, and potentially addressed. A previous paper also suggested that governance around treatments/procedures may be less regulated than administration of medication.^1^ This is an area that the governing bodies could consider how best to address. Looking to human sports medicine could be a useful source of specific agencies tasked with detection, policy, working with and coordinating stakeholders, and administering penalties (one such agency that demonstrates the all‐encompassing function is the World Anti‐Doping Agency).

Good regulation requires a good understanding of what is being regulated. The potential flaws in regulation and consequent illicit activity supports our earlier findings^1^ that it may be useful to clarify conceptually, and distinguish practically, between therapeutic (optimal), excessive, and performance enhancing veterinary provision. Defining the line between appropriate proactive veterinary intervention and excessive prophylactic veterinary intervention seems important in equine sports medicine. Furthermore, better understanding and separation between an intervention with a justifiable veterinary rationale and one aimed for performance enhancement appears necessary.

Regulation of veterinary procedures to performance horses can fall between the veterinary governing bodies and the sporting governing bodies. Only the RCVS has regulatory jurisdiction for the veterinary profession. The BEVA is the largest member organisation for equine veterinary surgeons in the UK. It produces extensive guidance and resources but is non‐regulatory. The sporting governing bodies are regulatory and can enforce regulations about veterinary procedures and veterinary medications, but should the onus and responsibility be left with the sporting organisations to debate and decide upon veterinary regulations? Veterinary and sporting organisations working together in a unified manner is required to enhance policy decisions. Furthermore, collectively working to understand the confidentiality issues which surround the reporting of concerning situations to governing bodies also appears necessary. It is hoped that understanding and acknowledging these pressures, will enable governing bodies to consider ways to further support veterinary surgeons.

### 4.1 Limitations

As this was the first research study to investigate ethical problems experienced by ESMVS, a questionnaire was developed to access a wide range of data. Although this technique did not provide in‐depth information about an individual veterinary surgeon's experiences, it did provide evidence of the range of issues and concerns facing this group. The aim was descriptive rather than definitive and has provided a foundation from which further research can be directed towards the issues highlighted in this study. Veterinary surgeons who had concerns about ethical issues in sports medicine, may have been more motivated to respond to the questionnaire. There is also the potential for response bias, whereby respondents may have provided answers they perceived were ethically desirable. Regarding the performance of the questionnaire, closed questions were answered by a higher number of respondents than the open‐ended questions, and many of the open‐ended questions were answered in the same way by individual respondents. The questionnaire was completed by 97 respondents which provides a good overview of ethical concerns. It is not clear what number of veterinary surgeons work specifically within the equine sports medicine discipline in the UK, but the membership of BEVA is approaching 3000. Further research using a qualitative method would complement this survey by gathering in‐depth information on the issues identified. Both are essential to inform ethical analysis and reasoning about the ethics of equine sports veterinary medicine.

## 5 CONCLUSION

This empirical research, which is the first to investigate ethical problems experienced by ESMVS, identified an array of contemporary concerns that are wide ranging and demand further study. The research has revealed areas that could pose reputational risk to equestrian sport and/or the veterinary profession. Governing bodies should consider how to improve support for veterinary surgeons facing ethical dilemmas, as for some these challenges lead to moral distress and may impact on recruitment and retention within the profession.

## FUNDING INFORMATION

Kate Allen obtained funding from a Bristol University Research Fellowship to undertake this study.

## CONFLICT OF INTEREST STATEMENT

The authors have declared no conflicting interests.

## AUTHOR CONTRIBUTIONS

**Kate Allen:** Conceptualization; methodology; software; data curation; formal analysis; funding acquisition; writing – original draft; writing – review and editing. **Mike King:** Supervision; writing – review and editing; methodology. **Lynley Anderson:** Supervision; writing – review and editing; methodology. **Siobhan Mullan:** Methodology; supervision; writing – review and editing.

## DATA INTEGRITY STATEMENT

Kate Allen had full access to all the data in the study and takes responsibility for the integrity of the data and the accuracy of data analysis.

## ETHICAL ANIMAL RESEARCH

This research study involves human participants only. Project approval was awarded by the University of Bristol Faculty of Health Sciences Research Ethics Committee (identification number 7563).

## INFORMED CONSENT

Participants consented to taking part by completion of the questionnaire.

### PEER REVIEW

The peer review history for this article is available at https://www.webofscience.com/api/gateway/wos/peer-review/10.1111/evj.14204.

## Supporting information


**Data S1:** Supporting Information.

## Data Availability

The data that support findings of this study are available from the corresponding author upon reasonable request: Open sharing exemption granted by the editor due to lack of provision in the informed consent process.

## References

[1] Allen K , Anderson L , King M , Mullen S . Competing interests at the heart of equine sports medicine ethics: a scoping review and thematic analysis. Equine Vet J. 2024;56(1):26–36. 10.1111/evj.13942 37163211

[2] American College of Veterinary Sports Medicine and Rehabilitation (ACVSMR). Available from: https://www.vsmr.org/

[3] Anderson LC , Gerrard DF . Ethical issues concerning New Zealand sports doctors. J Med Ethics. 2005;31:88–92. 15681672 10.1136/jme.2002.000836 PMC1734088

[4] Campbell ML . The role of veterinarians in equestrian sport: a comparative review of ethical issues surrounding human and equine sports medicine. Vet J. 2013;197:535–540. 23773811 10.1016/j.tvjl.2013.05.021 PMC3898908

[5] Mountjoy M , Rhind DJA , Tiivas A , Leglise M . Safeguarding the child athlete in sport: a review, a framework and recommendations for the IOC youth athlete development model. Br J Sports Med. 2015;49:883–886. 26084527 10.1136/bjsports-2015-094619 PMC4484277

[6] Anderson L . Writing a new code of ethics for sports physicians: principles and challenges. Br J Sports Med. 2009;43:1079–1082. 10.1136/bjsm.2008.051086 19474007

[7] Dvorak J , Baume N , Botré F , Broséus J , Budgett R , Frey WO , et al. Time for change: a roadmap to guide the implementation of the World Anti‐Doping Code 2015. Br J Sports Med. 2014;48:801–806. 10.1136/bjsports-2014-093561 24764550 PMC4033186

[8] Ardern CL , Grindem H , Ekås GR , Seil R , McNamee M . Applying ethical standards to guide shared decision‐making with youth athletes. Br J Sports Med. 2018;52:1289–1290. 29549146 10.1136/bjsports-2018-099183

[9] Campbell MLH . An ethical framework for the use of horses in competitive sport: theory and function. Animals. 2021;11(6):1725. 10.3390/ani11061725 34207809 PMC8230307

[10] Brown B , Cardwell JM , Verheyen KLP , Campbell MLH . Testing and refining the ethical framework for the use of horses in sport. Animals. 2023;13:1821. 10.3390/ani13111821 37889722 PMC10252045

[11] Yan T . Consequences of asking sensitive questions in surveys. Ann Rev Stat Appl. 2021;8:109–127.

[12] Anderson L . Contractual obligations and the sharing of confidential health information in sport. J Med Ethics. 2008;34(6):e6. 10.1136/jme.2008.024794 18757625

