# Supplementary material for: Ethical issues concerning UK veterinary surgeons practicing in equine sports medicine
Source: Equine Vet J. 2024 Jul 20;57(3):674–83. doi: 10.1111/evj.14204 (PMC11982412; doi:10.1111/evj.14204)
Supplement: Supplementary file 1 — Data S1: Supporting Information. [file EVJ-57-674-s001.pdf]

Equine Veterinary Journal BEVA

Questionnaire S1:

<u>Ethical issues concerning UK based veterinary surgeons practicing equine sports medicine.</u>

The ethics of sports medicine is an important area within human medicine and biomedical ethics, which thus far has received little attention within the veterinary field.

The aim of this questionnaire is to gather information on the ethical challenges that veterinary surgeons face during their equine sports medicine clinical practice.

This questionnaire forms the initial part of a research programme assessing equine sports medicine ethics, and is hoped will capture a broad view of the opinions of veterinary surgeons based in the UK.

This questionnaire is for veterinary surgeons who undertake some or all of their clinical veterinary activity within the field of equine sports medicine. For the purposes of this questionnaire equine sports medicine is considered to be a veterinary surgeons advising on 'the structural, physiological, medical, surgical and welfare needs of racehorses and sport horses and the restoration of normal form and function after injury or illness'. Thus, equine sports medicine covers all medical, orthopaedic, and surgical aspects of the veterinary care you provide. This questionnaire is specifically focused on horses (or ponies) who are being produced and trained for competition or racing.

The first part of the questionnaire is to collect some background information and the second part focuses on your opinions of any ethical issues facing equine sports medicine veterinary surgeons.

Completion of the questionnaire is entirely voluntary, and all responses are anonymous. By completing the questionnaire, your consent to take part in this study is implied. Once you have completed the questionnaire it is not possible to then withdraw from the study as the responses are anonymous and non-identifiable and cannot be retracted.

The questionnaire does take approximately 15 minutes to complete, and we are very grateful for your time and input. It has been developed by a collaborative research team which include practicing veterinary surgeons, specialists, ethicists, and BEVA committee members. The results/responses of the questionnaire may be used in publication/presentation and will be presented to BEVA to inform future strategy.

1. Approximately what proportion of your work is dedicated to clinical equine sports medicine provision*?
   <25%, 25-50%, 50-75%, >75%
   *For the purposes of this questionnaire equine sports medicine covers all medical, orthopaedic and surgical aspects of the veterinary care you provide to competition horses and racehorses (horses/ponies actively involved in racing, eventing, show jumping, dressage, polo, endurance, driving, vaulting, reining, para-equestrianism, showing).

2. Approximately what proportion of your sports medicine clinical service is undertaken in each of the following disciplines? (e.g., racing 40%, eventing 60%)
   Racing, eventing, show jumping, dressage, polo, endurance, driving, other (please specify)

3. Do you have any further post graduate clinical qualifications (e.g. Diploma, Certificate)?
   No, yes

If yes, Certificate, Diploma, Other (please specify)

4. Do you provide veterinary services at competitions/races?
   No, yes
   If yes:
   a. How often do you attend races to provide veterinary services? (more than once a month, 4-12 times per year, 1-3 times per year, less than annually)
   b. How often do you attend competitions (8 FEI disciplines* and polo) to provide veterinary services? (more than once a month, 4-12 times per year, 1-3 times per year, less than annually)

   * The eight FEI disciplines are: eventing, show jumping, dressage, endurance, carriage driving, vaulting, reining, para-equestrianism

5. Please rate the degree of responsibility that you feel towards the following stakeholders when you are providing sports medicine veterinary services, where 1 = 'I feel no responsibility towards this stakeholder' and 5 = 'I feel complete responsibility towards this stakeholder'
   Horse, owner, rider, trainer, yourself, your veterinary practice, RCVS, BEVA, BHA, FEI, other (please specify)
   RCVS: Royal College of Veterinary Surgeons; BEVA: British Equine Veterinary Association; BHA: British Horseracing Authority; FEI: Federation Equestre Internationale

6. What (if any) do you think are the ethical challenges facing veterinary surgeons who provide equine sports medicine services? (For the purposes of this questionnaire, an ethical challenge is a practice that compromises or might reasonably be expected to compromise horse welfare or the integrity of equestrian sport.) Please list or describe as many as you wish.
   Open text box

7. Can you briefly describe the circumstances of the most challenging situation you faced with regards to the provision of equine sports medicine veterinary services?
   Open text box
   a. How often does this situation arise?
      Only the once, rarely, a few times a year, monthly, weekly of more

8. The following questions are designed to identify factors which may lead to pressures or conflicts of interest for you when providing equine sports medicine veterinary services.
   a. Has the demand for a horse to compete/race ever caused any ethical issues for you as a veterinary surgeon?
      Never, rarely, a few times a year, monthly, weekly, daily
   b. Are you ever faced with a conflict of interest between your veterinary opinion of the horse’s best interest and the viewpoint of the owner/trainer
      Never, rarely, a few times a year, monthly, weekly, daily
   c. Are you ever faced with a conflict of interest where in order to retain a client you felt unable to act in the horse’s best interest
      Never, rarely, a few times a year, monthly, weekly, daily

d. Have you ever felt pressure from a veterinary surgeon within your practice to perform a veterinary service that you did not perceive to be ethically appropriate?
   Never, rarely, a few times a year, monthly, weekly, daily
e. Have you ever felt pressure from veterinary surgeons outside your practice to perform a veterinary service that you did not perceive to be ethically appropriate?
   Never, rarely, a few times a year, monthly, weekly, daily
f. Can you identify any other pressures or conflicts of interest that you face, with regards to sports medicine clinical service provision?
   Open text box

9. With regard to prohibited substances*, are there any medications or other substances that (i) are not prohibited which you think should be?
   Open text box

   With regard to prohibited substances*, are there any medications or other substances that (ii) are currently prohibited and you think should not be.
   Open text box

   *FEI: https://inside.fei.org/fei/cleansport/ad-h/prohibited-db
   *BHA: https://www.britishhorseracing.com/regulation/anti-doping-medication-control/prohibited-substances/

10. Sometimes veterinary surgeons are party to information that is highly sensitive. Have you ever become aware of:
    a. a horse being given a banned* substance
    b. a horse being given a controlled* medication too close to a competition
    c. a horse continuing in training against veterinary advice
    d. a horse competing with a known underlying disease process or injury that you perceived to be either likely to be significantly worsened by competing, and/or a significant detriment to welfare
    e. a horse undergoing a veterinary procedure not permitted under RCVS regulations
    f. a horse undergoing a veterinary procedure not permitted by the sport's governing body (e.g. BHA, FEI)
    g. an owner/trainer themselves administering a medication to a horse without specific veterinary guidance
    h. an owner/trainer undertaking other prohibited activities (such as limb hyper/hyposensitisation)

    No, once, occasionally, frequently
    If you answered yes (once, occasionally, frequently), to any of the above questions did you report this to any governing body
    Open text box
    *For the purposes of this questionnaire the following definitions should be used:
    Banned substances – substances deemed by the governing body to have no legitimate use in the competition horse and/or have a high potential for abuse. They are not permitted for use in the competition horse at any time.

Controlled medications – substances deemed by the governing body to have therapeutic values and/or be commonly used in equine medicine. However, controlled medications have the potential to affect performance and/or be a welfare risk to the horse and they should not be present during competitions.

11. Please list any permitted* veterinary treatments or procedures (medical or surgical) for competition/racehorses that you consider ethically unacceptable? *permitted under RCVS regulations
    Open text box

12. Please list any permitted veterinary treatments or procedures that you think are used excessively within equine sports medicine, such that they may not be in the horse's best interest. *permitted under RCVS regulations
    Open text box

13. During a competition/race the duties and obligations of veterinary surgeons are well-defined by regulatory bodies.
    Strongly agree, agree, neutral, disagree, strongly disagree
    RCVS, BHA, FEI, HPA, Other (please specify)
    RCVS: Royal College of Veterinary Surgeons; BHA: British Horseracing Authority; FEI: Federation Equestre International; HPA: Hurlingham Polo Association

14. Between competitions/races the duties and obligations of veterinary surgeons are well-defined by regulatory bodies (strongly agree, agree, neutral, disagree, strongly disagree)
    RCVS, BHA, FEI, HPA, Other (please specify)
    RCVS: Royal College of Veterinary Surgeons; BHA: British Horseracing Authority; FEI: Federation Equestre International; HPA: Hurlingham Polo Association

15. Which equine sports medicine ethical challenge (if any) do you think is the highest priority for the industry to address?
    Open text box

16. Please list any elements of equine sports medicine that you believe to pose a reputational risk to equestrian sport i.e. elements that may cause significant public concern.
    Open text box

17. Please list any elements of equine sports medicine that you believe to post a reputational risk to the veterinary profession
    Open text box

18. Please list the areas you feel would be highest priority for further research aimed at improving the welfare of competition/racehorses?
    Open text box

19. In light of the aims of this questionnaire (as stated in the introduction), are there any other issues that are of concern to you as an equine sports medicine veterinary surgeon?
    Open text box
